# A Comprehensive Review of Prognostic Factors in Patients with Gastric Adenocarcinoma

**DOI:** 10.3390/cancers15051628

**Published:** 2023-03-06

**Authors:** Styliani Mantziari, Penelope St Amour, Francesco Abboretti, Hugo Teixeira-Farinha, Sergio Gaspar Figueiredo, Caroline Gronnier, Dimitrios Schizas, Nicolas Demartines, Markus Schäfer

**Affiliations:** 1 Department of Visceral Surgery, University Hospital of Lausanne, Rue du Bugnon 46, 1011 Lausanne, Switzerland; 2 Faculty of Biology and Medicine, University of Lausanne (UNIL), 1015 Lausanne, Switzerland; 3 Oeso-Gastric Surgery Unit, Department of Digestive Surgery, Magellan Center, Bordeaux University Hospital, 33600 Pessac, France; 4 Faculty of Medicine, Bordeaux Ségalen University, 33000 Bordeaux, France; 5 First Department of Surgery, National and Kapodistrian University of Athens, Laikon General Hospital, 11527 Athens, Greece

**Keywords:** gastric cancer, adenocarcinoma, prognosis, survival

## Abstract

**Simple Summary:**

Gastric adenocarcinoma remains associated with a poor prognosis despite recent therapeutical advances. As diagnosis is frequently made at an advanced stage, long-term outcome is dismal. Individualized identification of factors associated with poor prognosis allows more precise survival prediction and, in some cases, to propose targeted treatment through individualized precision medicine. This review aims to highlight the prognosis determinants of these tumors and potential therapeutical impact from the available literature.

**Abstract:**

Gastric adenocarcinoma remains associated with a poor long-term survival, despite recent therapeutical advances. In most parts of the world where systematic screening programs do not exist, diagnosis is often made at advanced stages, affecting long-term prognosis. In recent years, there is increasing evidence that a large bundle of factors, ranging from the tumor microenvironment to patient ethnicity and variations in therapeutic strategy, play an important role in patient outcome. A more thorough understanding of these multi-faceted parameters is needed in order to provide a better assessment of long-term prognosis in these patients, which probably also require the refinement of current staging systems. This study aims to review existing knowledge on the clinical, biomolecular and treatment-related parameters that have some prognostic value in patients with gastric adenocarcinoma.

## 1. Introduction

Gastric cancer (GC) is presently the 5th most common cancer diagnosis worldwide, corresponding to 5.6% of all new cancer cases in 2020 (>1,000,000 patients) [1]. GC displays a predominance in the male gender, with particularly high endemic areas in Asia, where its incidence reaches up to 29.5/100,000 in Japan and Korea [2]. Overall, 75.3% of all GC diagnoses affect Asian populations [3]. Although the pathogenesis of gastric cancer remains unclear, chronic gastritis is believed to participate in cellular changes, leading to malignant transformation. This situation is often encountered in patients with autoimmune gastritis, and H. pylori infections [4]. Over the last 20 years, a progressive decrease in distal gastric cancer has been observed, in relation to H. pylori screening and eradication programs in endemic countries. On the other hand, we can see an increase in proximal gastric and gastro-esophageal junction (GEJ) lesions, in relation to obesity and poorly controlled gastro-esophageal reflux disease (GERD) [5]. Other risk factors, such as a diet with a high intake of salt [6] and smoking [7], have been identified.

Along with changing epidemiology, several aspects of staging, therapeutic options and insight into tumor biology have evolved over the years. Thus, the traditional view of patient prognosis predicted mainly by baseline tumor-stage is outdated, and all of these parameters need to be taken into account to offer the best possible counseling and tailored treatment options to patients with GC. This study aims to provide an updated review of the existing knowledge on the clinical, biomolecular and treatment-related parameters that affect the prognosis of patients with gastric adenocarcinoma.

## 2. Materials and Methods

A comprehensive literature review was performed in the MEDLINE library through Pubmed and EMBASE to identify articles focused on the prognostic tools and predictive factors of long-term survival in patients with GC. Only full-text articles were included in the study, with a time-span limit from 1995 to 2022, with no formal language restrictions. Reference lists of included articles were hand-searched to identify further relevant studies that may have been missed by the search algorithm.

## 3. Results

### 3.1. Predictive Tools of Long-Term Prognosis in Gastric Cancer Patients

#### 3.1.1. The TNM/UICC Staging System

**Figure 1:**
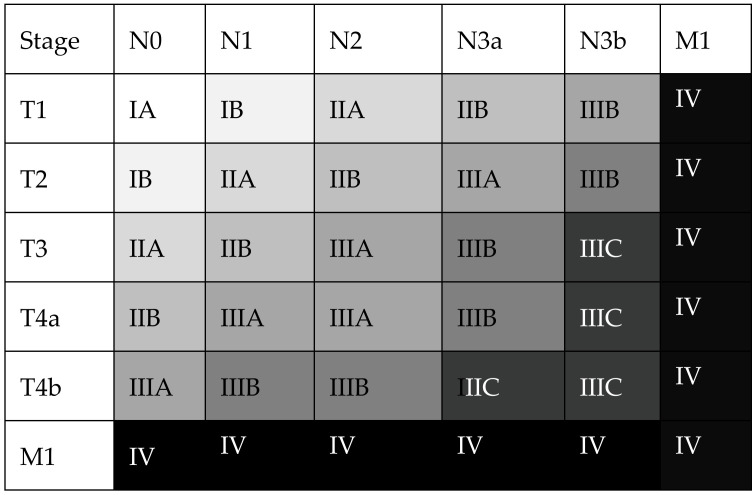
The 8th edition of the TNM/UICC staging system for gastric cancer.

The TNM (Tumor, Nodes, Metastasis) classification was first described in the 1940s by Dr Pierre Denoix to offer a standardized description of the parietal invasion of the tumor (T stage), the locoregional lymphatic invasion (N stage) and the presence of distant metastases (M stage). Its latest 8th edition, published in 2017, is the current standard of solid tumor staging [8] (Figure 1). The main difference with the previous version lies in the subdivision of stage N3 in N3a (7–15 positive lymph nodes) and N3b (≥16 positive lymph nodes). This subdivision highlights that in case of massive lymphatic invasion, overall prognosis approximates the one of metastatic disease. Recent studies report an excellent prognostic value of the 8th edition of the TNM staging system, superior to the previous edition concerning stages IIIB and IIIC [9,10,11,12].

Legend: The UICC/TNM tumor stage (I–IV) is determined by the depth of wall invasion (T), lymphatic spread (N) and the presence of distant metastases (M). Adapted from [8].

Large-scale studies from the American National Cancer Database (NCDB) [10] and the American Surveillance, Epidemiology and End Results (SEER) registry [12] confirm an excellent discriminatory capacity of the 8th TNM classification, with overall survival decreasing inversely with tumor stage. It is, however, remarkable that, even if the validation population was similar in these two studies, estimated 5-year survival differs for the same TNM stage; it is reported as being between 50.8% and 59.3% for stage IIA, 35.3–46.4% for stage IIB and 20.5–30.5% for stage IIIA (Table 1).

Creation and validation of the TNM system is based on a large series of registry studies, and thus is exposed to large variability in surgical techniques (resection radicality, lymph node dissection field) and perioperative treatment plan (e.g., use of perioperative chemotherapy). In the two American series mentioned above, radicality of lymph node dissection is inferior to current standards (>15), with a median of only 2 [10] and 13.5 [12], respectively, whereas no information is available on post-operative chemotherapy in the SEER registry [12]. When the same staging system was validated in an Asian series by Lu et al. [13], 5-year survival was superior, even for advanced stages (5-year survival 27.5% vs. 8.3% for stage IIIC tumors) (Table 1). Indeed, apart from the differences in lymph-node dissection, (median of 2 [10] versus 32 resected lymph nodes [13]), a higher rate of patients with adjuvant treatment was observed in the Asian series (57.6% vs. 50%) [13]. Graziosi et al., in a European series, reported survival rates largely inferior to the Asian data [11].

The variability in treatment strategies between Eastern and Western populations, along with other poorly understood mechanisms, highlights how much caution needs to be taken when using the TNM stage for individual patient prognostic estimation and counseling.

#### 3.1.2. Alternative Prognostic Tools beyond the TNM

**Figure 2:**
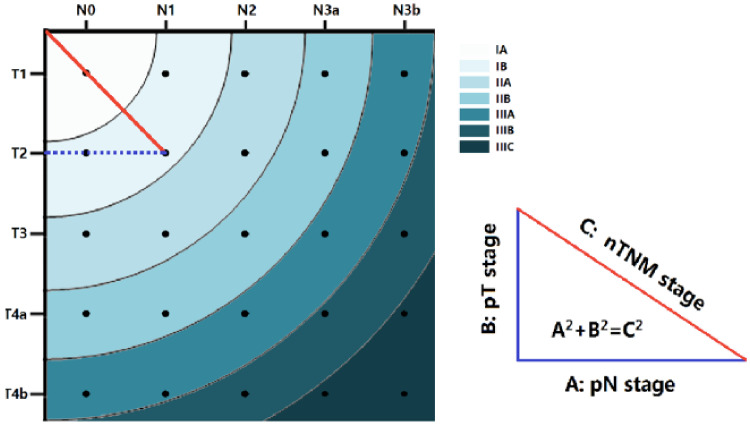
The ‘metro ticket’ prognostic tool for gastric cancer; the more we move further from the center, the worse the prognosis [13].

To compensate for the above-mentioned TNM/UICC shortcomings, some newer staging systems have appeared in the oncologic landscape. The “metro-ticket” model [13] uses the same definitions for pT and N as the 8th TNM version, while the tumor stage as a whole is defined using the distance to the point ‘0′ of x and y axis (Figure 2). The further you get away from point ‘0′, the greater the stage and the worse the prognosis (in analogy to metro-ticket fees; the further away from the center, the more expensive it becomes). With this model, 5-year survival was estimated to 94.8% for stage IA, 88.0% for stage IB, 80.1% for stage IIA, 67.9% for stage IIB, 54.4% for stage IIIA, 35.9% for stage IIIB and 27.4% for stage IIIC. Two large series (one Asian and one American) externally validated this system and found a comparable prognostic value with the 8th TNM edition, with even superior discriminatory value for stages IB and IIA [13,14]. However promising, the ‘metro ticket model’ closely follows the TNM paradigm, trying to predict long-term survival on the sole basis of baseline tumor stage.

**Figure 3:**
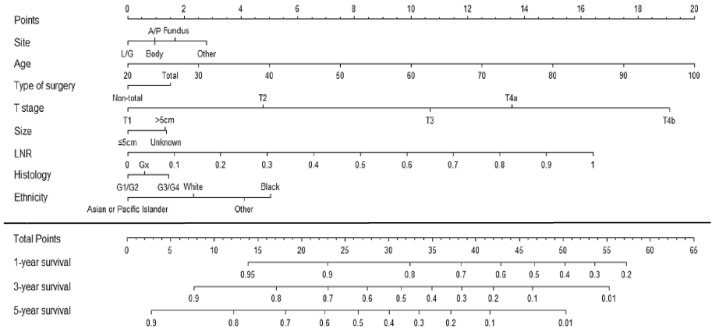
Predictive nomogram of survival after curative resection of gastric cancer, from Lu et al. [12]. Footnote: LNR = Lymph Node Ratio. Each of the included variables attributes a number of points according to the tumor and patient’s characteristics. The total addition of points provides the predicted percentage of 1-, 3- and 5- year survival rate.

Similarly, Lu et al. [12] recently developed a predictive nomogram taking into account not only stage, but also other clinico-pathological characteristics: age, ethnic group, tumor site, surgical resection technique, T stage, tumor size, lymph node ratio and differentiation grade (Figure 3). The prognostic value of this nomogram (C-index) was similar to the 8th edition of TNM for patients with a lymph node yield of > 15 nodes, and superior for patients with suboptimal lymph node dissection (≤15 nodes). This model was developed within a large Asian series and validated in an American registry cohort (SEER) [14]. Even if its external validity still needs to be proven in other populations, it was the first to integrate patient features, treatment characteristics and tumor biology among the prognostic factors. Since then, other authors have proposed various nomograms predictive of survival in GC patients, based on clinicopathological data (pre- and post-operative), biological markers (e.g., ASAT/ALAT [15]) or gene sequencing [16,17]. Some prognostic tools have been developed to target specific populations, such as elderly patients [18] or patients with signet ring cell carcinoma [19]. With the multitude of prognostic tools available, no single model, however sophisticated, can be considered reliable enough to predict individual patient prognosis.

### 3.2. Biological Features of Gastric Cancer and Their Impact on Long-Term Prognosis

In recent years, some further insight has been gained into the inherent biologic features of the disease, potentially influencing long-term prognosis.

#### 3.2.1. Ethnicity

As illustrated in Table 1, the long-term prognosis of patients with GC changes significantly depending on their country of origin, even for similar disease stages. Part of the explanation for this may lie in the detection of the disease in earlier stages in endemic countries, such as Japan and Korea, due to the population screening programs. However, it has been demonstrated that even for advanced stages, prognosis remains more favorable for Asian patients (Table 1).

Treatment-related differences may further account for this prognostic difference. After surgical resection, reported 5-year survival (all-stages included) reaches 60% in Japan/Korea, whereas it does not exceed 20% in the USA/UK [20,21,22,23]. Recently, Yamada et al. comparatively analyzed two series: one from the UK (*n* = 46) and one from Japan (*n* = 465) ([20]. The Japanese series included earlier cancer stages (57% stage I vs. 14%, *p* < 0.001), more (oligo-)metastatic patients at diagnosis (9% vs. 3%, *p* < 0.001) and more diffuse type tumors (54% vs. 13%) [20]. The UK series had more positive resection margins (14% vs. 5%, *p* < 0.001) and suboptimal lymph node dissection (<15 nodes) in 39% vs. 8% of cases (*p* < 0.001). To adjust for these differences, the authors performed a survival analysis after propensity score matching; even so, 5-year OS was 69% in Japan and 52.2% in the UK (*p* < 0.001), whereas cancer-related 5-year survival was 75.3% vs. 64.9%, respectively (*p* = 0.003). In the Japanese series, age > 65 years and stage pT4 and pN2-3 were independently associated with long-term mortality, whereas in the UK, in addition to those factors, localization of the tumor to the entire stomach, synchronous pancreatectomy, R1 resection and <15 lymph node dissection were identified as poor prognostic factors [20]. These data suggest that the impact of the ethnic origin on survival cannot only be attributed to the difference in stage and radical surgery. Strong et al. [24], in a comparative study between USA and Korean series, also reported earlier lesions (62% stage I vs. 49%) and more radical lymph node dissection in Korea (only 3% of patients with <15 lymph nodes vs. 22% in USA). Once again, multivariable analysis, adjusting for these factors, demonstrated more favorable OS for Korean patients (HR = 1.3, 95%CI 1.0–1.7, *p* = 0.05). Even if the rigorous scientific methodology of these studies allows excluding differences in stages and surgical radicality as the only explanation for survival differences, some potential bias can be suspected. For example, stage migration potentially associated with a lymph node yield of < 15 nodes can under-estimate tumor stage in western patients, with an impact on the indication for a post-operative systemic treatment [25].

Still, treatment variabilities do not seem to totally explain the prognostic differences between Eastern and Western series, suggesting the implication of inherent biologic differences related to ethnicity. In a study of the American register SEER, Asian patients treated in the USA had a clear survival advantage compared to Caucasian patients treated with the same practices, especially for early stages [26]. These data were also confirmed in an NCDB series of >4000 patients, comparing White, Black and Asian Americans operated on for GC in the USA [27]. Indeed, Asian-American patients were found to have greater lymph node yield, decreased lymph node ratio, lower postoperative mortality, and more favorable long-term survival compared to White or Black Americans [27].

#### 3.2.2. Sex-Related Differences

In recent years, sex-related differences in cancer treatment and prognosis are becoming apparent. Significant variations have been reported in the choice of treatment modalities and their toxicity among oesophagogastric cancer patients treated with curative intent [28]. Female patients seem to experience higher toxicity from systemic chemotherapy, which cannot solely be attributed to lean body mass differences between men and women [29]. In the recently published report from the Dutch nationwide cohort, female patients with gastric cancer had better postoperative outcomes, but a significantly lower 5-year relative survival compared to males (49% vs. 56%, RER = 1.31, 95% CI 1.09–1.58, *p* = 0.004) [30].

#### 3.2.3. Histological Subtype

Lauren’s classification, established in 1965, subdivides gastric adenocarcinoma into diffuse and intestinal types [31]. Tang et al. confirmed a prognostic value of this classification, analyzing a series of 20,000 patients from the SEER database; they demonstrated that diffuse type, and especially lesions >T1 and >2 cm, is independently associated with worse cancer-specific survival [32].

Approximatively 5% of gastric cancers present with a diffuse gastric wall infiltration, named “linitis plastica”. This diffuse type, according to Lauren, is often associated with this aggressive subgroup, with a high risk for extended submucosal infiltration and distant micro-metastases. Signet-ring cells (SRCs) (otherwise called independent or poorly cohesive cells) are encountered in approximately 25% of gastric cancer patients in the USA [33]. They are more often found in the distal part of the stomach, in young female patients, and present a particularly aggressive biological behavior, with a metastatic stage upon diagnosis in 50% of patients [33]. Although SRC histology has traditionally been associated with poor prognosis, some discordant data have been published in the literature. A large-scale American series of >10,000 patients suggested similar survival outcomes to non-SRC adenocarcinoma, when stratified by stage: 30 vs. 40 months for stage II, 19 vs. 20 months for stage III, 6 vs. 7 months for stage IV [33]. Moreover, recent meta-analyses have demonstrated that SRC histology is not necessarily an aggravating prognostic factor for early stages of the disease, but it remains associated with worse prognosis for advanced stages [34,35].

SRC lesions are known to be less sensitive to chemotherapy than non-SRC adenocarcinoma. Messager et al., in a French series of 924 patients, reported that traditional 5-FU-platin-based chemotherapy did not offer a survival or R0 resection benefit in SRC patients [36]. However, the FLOT regimen, the current chemotherapy standard in many centers, has demonstrated its efficacy, even in this challenging histological subgroup [37].

#### 3.2.4. Tumor Micro-Environment, Genetic Phenotypes

In recent years, significant advances have been made regarding the better understanding of GC and the host immune response. With genomic sequencing, some new classifications were proposed.

First, The Cancer Genome Atlas Project (TGCA, 2014) identified four molecular subtypes of gastric adenocarcinoma: chromosome instability (CIN, 50%), micro-satellite instability (MSI-H, 22%), genomically stable (GS, 20%) and tumors associated with an Epstein–Barr Virus infection (EBV, 9%) [38]. The CIN subtype is principally encountered in proximal or junctional lesions, (65%), associated with RTK-RAS mutations, whereas the EBV type is mostly found in the gastric body or antrum (62%), associated with PD-L1/2 overexpression. Of note, no significant difference of genetic subtype was found between Occidental and Asian patients [38]. The prognostic significance of this subdivision has not yet been established in clinical practice. However, EBV-tumors were found to have a better prognosis and GS a poorer one, whereas response to adjuvant chemotherapy was also dependent on the subtype, with more favorable outcomes for CIN-patients [39].

Second, the Asian Cancer Research Group (ACRG, 2015) also identified four molecular subtypes: MSI (microsatellite unstable), MSS/EMT (microsatellite table, epithelial-mesenchymal transition), MSS/TP53+ (tumor protein 53 aberrant) and MSS/TP53- (tumor protein 53 inactive). The MSS/EMT subtype was found to have the worst prognosis, while MSI had the best one; TP53-subtypes have intermediate prognosis [40].

Pietrantonio et al., in a recent metanalysis, found the MSI-H subtype in 7.8% of all GC patients [41]. This group, when compared to those with micro-satellite stability (MSS), demonstrated a more favorable 5-year OS (77.5% vs. 59.3%) and 5-year DFS (71.8% vs. 51.3%), with MSI-H status being independently associated with better long-term survival. Moreover, MSI-H patients did not show a significant survival benefit after treatment with traditional chemotherapy; however, such a benefit was observed when targeted PD-1/PD-L1 blockade was used [42]. Up to now, MSI-H status is the only genetic subtype with some prognostic and therapeutic implications, whereas the other subtypes have not demonstrated any particular prognostic value per se. 

When trying to understand prognostic variations among Eastern and Western populations, genomic differences might seem to be a potential explanation. Indeed, alterations in large sequences, especially b-cadherin, were identified more often in Caucasian than in Afro-American or Asian patients, although no significant association with patient prognosis was found [43]. Park et al. suggested a prognostic difference between Asian and Caucasian patients related to the variability of expression of the vascular endothelial growth factor (VEGF-A) [44]. These scarce data illustrate that genetic variability of GC in different ethnic groups is not yet completely elucidated. Thus, the relative prognostic advantage of Eastern populations discussed above can only be partially explained with available evidence [42].

Some previous data suggested better long-term prognosis for patients with distal stomach tumors compared to those with proximal ones, even for identical stages [45]. Although the exact reason for this prognostic difference remains unclear, genetic variability of these two locations of GC (more CIN subtype for proximal versus more EBV-related subtype for distal lesions) could offer an explanation. However, these preliminary data need further validation before being considered among the prognostic factors of the disease.

### 3.3. Improvement of Gastric Cancer Prognosis over Time; Evolution of Treatment Strategies for Resectable Disease

The evolution of treatment options for GC patients over time and its impact on prognosis cannot be overlooked. Systemic chemotherapy, both preoperative and adjuvant, is now widely performed, except for very early stages of the disease. Multimodality management is key to improving patient outcomes, with standards of oncologic surgery as well as chemotherapy efficacy improving over time.

#### 3.3.1. Surgery

In the past, when surgery was the only curative treatment available, 5-year overall survival was estimated at 19%, and cancer-related 5-year survival at 26%, even for curable stages [45]. Survival did not exceed 50% for stage I, 29% for stage II, 13% for stage III and barely 3% for stage IV [45]. Even in the more recent MAGIC trial, patients with locally advanced GC undergoing surgery alone experienced a 5-year OS survival of only 23% [23]. Although one may criticize the quality of surgical resection in this trial, it does reflect the treatment standards available at that time, which have considerably evolved since.

The type of surgical resection can influence prognosis, depending on tumor location. Total gastrectomy is suggested for proximal stomach tumors, whereas partial gastrectomy for lesions of the distal 2/3 of the stomach offers similar survival results, when negative margins (R0) are achieved [46]. The optimal resection margin for GC was traditionally considered as >5 cm, and even 8 cm for SRC tumors [47]. However, these margins are sometimes impossible to obtain, but have also failed to demonstrate a survival advantage or decreased recurrence risk compared to closer margins, if an R0 resection is obtained [47]. More recent data suggest a survival advantage for resection margins >3 cm in the case of early tumors (stage I), which increase for more advanced stages [48].

The radicality of lymph node dissection is a major prognostic factor but also a quality indicator for GC surgery. The current standard requires a D2 lymph node dissection sparing the spleen, with at least 16 resected lymph nodes on the operative specimen [8]. The exact mechanism of lymph node dissection on improving patient prognosis is not completely elucidated. On the one hand, it could be related to the suppression of the lymphatic network containing metastases. In addition, it could also be associated with the effect of stage migration of patients with an inadequate lymph node dissection, underestimating the need for systemic adjuvant treatment. Currently, there are few data demonstrating a survival advantage of a D2 dissection (involving the coeliac trunk main arteries) when compared to a dissection limited to perigastric lymph nodes (D1). Fifteen-year results of the Dutch randomized trial D1D2 did not demonstrate an OS benefit of D2 dissection, but cancer-specific mortality was higher after D1 dissection [49]. Mocellin et al., in a recent meta-analysis, reported an OS and DFS similar after D1 and D2 lymph node dissection. Cancer-related survival was improved in the D2 group with, however, increased post-operative mortality, limiting the OS benefit for patients. Finally, no prognostic advantage was demonstrated for a super-extended lymph node dissection (D3) when compared to D2 lymphadenectomy [50].

#### 3.3.2. Systemic Chemotherapy

The widespread use of chemotherapy has played a pivotal role in improving prognosis for patients with gastric cancer during recent years.

The MAGIC trial, published in 2006 [23], demonstrated a significant survival advantage of perioperative ECF chemotherapy (Epirubicin, Cisplatin and 5FU) compared to surgery alone for resectable GC; 5-year OS increased from 23% to 36.3%, and median DFS from 12 to 19 months, respectively [23]. Even if the quality of surgical resection and postoperative treatment completion rate were strongly criticized, it has played a pivotal role to the recent improvement of patient prognosis through systemic treatment. A perioperative 5FU-Cisplatin regimen compared to surgery alone in a French trial also offered a significant survival benefit for the multimodal treatment group, with a 5-year overall survival of 38% compared to 24% for surgery alone (*p* = 0.02) [51]. More recently, the landmark FLOT trial demonstrated a survival benefit of the FLOT regimen (5-FU, Leucovorin, Oxaliplatin, Docetaxel) compared to ECF, with an increase of median OS from 35 to 50 months (*p* = 0.012) and DFS from 18 to 30 months (*p* = 0.0036) [37]. These results were confirmed in a large series of British patients that also demonstrated similar rates of perioperative adverse events and systemic treatment compliance between the two regimens [52].

The CLASSIC trial, based on a large Asian series, found a prognostic advantage for patients treated with surgery and adjuvant chemotherapy (capecitabine-oxaliplatine) compared to surgery alone (3-year disease-free survival 74% vs. 59%, *p* < 0.001) [21]. Thus, post-operative chemotherapy offers a survival advantage for patients with locally advanced tumor without neoadjuvant treatment and is considered as the treatment of choice in Asia. More recently, the phase III PRODIGY trial compared perioperative chemotherapy (DOS–Docetaxel, Oxaliplatin, S-1) to surgery with adjuvant treatment [53], demonstrating a disease-free survival advantage in the group with neo-adjuvant treatment (HR 0.70; 95% CI 0.52 to 0.95).

#### 3.3.3. External Beam Radiation

Post-operative chemoradiotherapy is a widely used treatment option in the USA. The MacDonald trial in 2001 suggested a survival benefit for gastric and junctional adenocarcinoma treated with surgery and adjuvant radiochemotherapy (5-FU leucovorin+45Gy) vs. surgery alone, with a median survival of 36 and 27 months, respectively (*p* = 0.005) [22]. Ten-year follow-up results of this study confirmed that the benefit is maintained in the long term, both in terms of systemic and locoregional relapse, and overall survival [54]. In this trial, the authors recognized high rates of suboptimal lymph node dissection in the USA that can influence outcomes, obviating the benefit of systemic treatment to improve long-term prognosis.

#### 3.3.4. Advanced Disease, Peritoneal Carcinomatosis

One subgroup of GC patients who has seen their prognosis greatly improve in recent years are stage IV patients with isolated peritoneal carcinomatosis. GC presents with peritoneal metastases in up to 20–30% of cases [55], while among all patients with peritoneal carcinomatosis, 14% are of gastric origin [56]. They were, until recently, only eligible for palliative chemotherapy, with 5-year survival rates not exceeding 5% and median survival of 9 months [10,11]. Nowadays, selected patients with localized peritoneal stage IV disease are considered for additional treatment options, including local intraperitoneal chemotherapy and cytoreductive surgery.

Pressurized Intraperitoneal Aerosol Chemotherapy (PIPAC), with a platin-combination regimen, may be proposed alone or in combination with systemic chemotherapy, to halt local disease progression and control refractory ascites, with encouraging results in terms of histologic response and median survival reaching 13–15 months [57,58]. Indeed, intraperitoneal chemotherapy has proven its efficacy in local tissue penetration of peritoneal metastases, which are notoriously resistant to systemic chemotherapy. Although PIPAC remains a palliative-intent treatment in the majority of cases, it has demonstrated its safety and acceptable toxicity profile, even combined with several cycles of systemic chemotherapy [59].

Hyperthermic Intraperitoneal Chemotherapy (HIPEC) combined with surgery represents a more aggressive option, offered with curative intent with the aim of obtaining complete cytoreduction. The recently published CYTO-CHIP study from France has demonstrated a significant survival benefit for patients treated with complete cytoreductive surgery (CRS) and HIPEC compared to those with CRS alone, especially for limited peritoneal disease with a carcinomatosis index (PCI) < 7 [60]. In this large-scale multicentric trial, CRS/HIPEC patients demonstrated superior median OS, with 18.8 versus 12.1 months, 5-year OS with 10.8% versus 6.4% (*p* = 0.005), and 5-year recurrence-free survival with 5.9% and 3.8%, *p* = 0.001) [60].

Although more robust data are needed to optimize patient selection for intraperitoneal chemotherapy, the promising results of the above-mentioned strategies have already set a new landscape to improve prognosis in patients with gastric carcinomatosis.

#### 3.3.5. Targeted Therapies

A better understanding of tumor biology and the molecular mechanisms of gastric cancer has allowed some considerable advances in patient care over the years. Currently, targeted agents (otherwise known as immunotherapy) are widely used with very promising results for different types of malignancies, including gastric cancer. The tumor micro-environment, expression of membrane protein growth factors and host immune cell infiltration are of paramount importance in this context.

Human epidermal growth factor receptor (HER2) overexpression, found in 7–34% of gastric adenocarcinoma patients, is currently a decisive element in disease treatment, as these tumors are good responders to the monoclonal antibody trastuzumab [61]. The landmark ToGA trial, including principally metastatic gastric cancer patients, demonstrated a survival benefit for patients treated with a combined regimen (chemotherapy + Trastuzumab) compared to chemotherapy alone (median OS 13.8 months versus 11.1 months, *p* = 0.0046) [61]. Although the absolute survival benefit may seem clinically insignificant, it needs to be kept in mind that this was a group of mostly metastatic, heavily pretreated patients, for whom few or no therapeutic options were available.

To decrease the toxicity of these treatments and improve their efficacy, new agents were developed, the antibody-drug conjugates (ADC). These treatments combine monoclonal antibodies with cytotoxic molecules. They seem to have promising results in advanced gastric cancer, although further studies are needed to optimize their composition and identify new targets [62]. An ongoing clinical trial is assessing Chimeric Antigen Receptor (CAR) T cell therapy targeting Claudin 18.2 in GC [63].

Another membrane protein complex, PD-1/PD-L1, is a well-known immune mediator, ‘blunting’ the immune system reaction and allowing malignant cells to escape from the host’s immunosurveillance. Targeted anti-PD-1 agents such as pembrolizumab have shown very promising results in this context. Chao et al. performed a post-hoc analysis of three randomized trials to assess the benefit of the anti-PD-1 monoclonal antibody pembrolizumab in patients with advanced gastric cancer [64]. Among all patients, a 2-year OS rate of 24% (95% CI 19–30) was observed when pembrolizumab was combined with standard chemotherapy, whereas it increased to 65% (95% CI 38–82) for the subgroup with microsatellite instable (MSI) tumors, who had a bare 26% 2-year OS for similar stages of the disease [42]. The recently published Checkmate-649 trial [65], including metastatic patients with no previous treatment, also showed a significant survival benefit of the anti-PD-1 monoclonal antibody nivolumab, with a median OS of 14.4 months (95% CI 13.1–16.2) versus 11.1 months (95% CI 10–12.1) after traditional cytotoxic chemotherapy.

The above-mentioned studies, among multiple other ongoing trials, illustrate the paradigm shift of recent years towards adapting individual patient treatment to tumor biology. This approach, known as precision medicine, offers very promising results for gastric cancer patients, even in advanced, previously untreatable stages, of the disease.

## 4. Discussion

Gastric cancer has traditionally been associated with an aggressive biological behavior and dismal prognosis, even in potentially curative stages of the disease. In recent years, not only more elaborate prognostic tools have been developed to guide patient counselling and management, but also treatment options and understanding of the biologic basis of the disease have significantly evolved, with a subsequent improvement of prognosis.

In the 1980s, 5-year OS by stage reached 50% for stage I, 29% for stage II, 13% for stage III and barely 3% for stage IV [45]. Currently, respective survival rates have increased to 63–94% for stage I, 51–68% for stage II, 20–33%% for stage III and 5% for stage IV [8,10,11,12,13]. Current treatment standards, with the wide use of neoadjuvant/adjuvant chemotherapy, radical oncologic surgery with D2 lymph node dissection and the adjunct of targeted treatments for advanced disease, has led to significant improvements in survival. The advent of technical advances, such as minimally invasive surgery, has improved the quality of surgical specimens’ precision of lymphadenectomy and atraumatic tissue handling, influencing overall prognosis and reducing postoperative morbidity. In patients with metastatic disease, important leaps forward have been observed in recent years. Median survival, estimated at around 4 months when treated only with the best supportive care, has increased to around 12 months after systemic chemotherapy [66], and up to 18.8 months when metastatic disease is limited to the peritoneal cavity and treated with complete cytoreduction and HIPEC [60].

Although the overall improvement of prognosis may be largely attributed to the above-mentioned recent advances of surgical and systemic treatment of gastric cancer, the present review has identified several other factors related to long-term outcomes. The role of patient ethnicity in gastric cancer prognosis has been documented for several years [25,67]. Apart from some obvious differences in baseline stage and treatment modalities between Eastern and Western populations, inherent biologic variability might play a role in patient prognosis. Although not entirely elucidated up to this day, the biomolecular background of gastric cancer may provide some insight into the exact role of patient ethnicity on long-term prognosis.

Similarly, some significant treatment and prognosis-related differences have been reported, related to patients’ sex. Gender medicine is rapidly evolving and is expected to provide some further evidence on the observed differences in treatment and prognosis of male versus female patients. Although a prognostic advantage has been reported for male gastric cancer patients in the Dutch nationwide study, a clear physiopathologic explanation cannot be provided now [30]. Thus, caution needs to be taken when integrating patient sex and ethnicity in the prognostic tools for gastric cancer, especially when it comes to individual patient counselling. Indeed, these parameters need to be taken into careful consideration among a multitude of other factors, including staging, biology and available treatment options, to avoid introducing further bias and thus amplify the observed prognostic difference.

## 5. Conclusions

Gastric adenocarcinoma, despite having a dismal overall prognosis, has seen some substantial improvement in recent years. Disease course is closely related to tumor stage (TNM/UICC 8th edition), but also to other clinico-biological factors that must be considered. Ethnicity is a major prognostic factor, with Asian patients having more favorable outcomes than Occidental patients for the same tumor stage. Radicality of surgery, with R0 margins, and D2 lymph node dissection with >15 nodes, with perioperative radiochemo/chemotherapy offer better prognosis than surgery alone for locally advanced tumors. In the last few decades, genomic sequencing of gastric adenocarcinoma highlighted different genetic subtypes. These genetic subtypes have as yet no clear influence on prognosis, except for the MSI-H subtype, which appears to have more favorable outcomes than MSS lesions and responds better to systemic immunotherapy. Targeted agents against tumors over-expressing HER2 and PD-L1/2 have shown promising results in advanced GC, and their efficacy is being tested in ongoing trials evaluating this impact in earlier treatment stages.

## Figures and Tables

**Table 1:** Reported prognosis of 5-year overall survival by stage, for patients with gastric adenocarcinoma.

Tumor Stage (8th TNM ed., [8])	American NCDB Registry [10]	5-Year OS (%)	Median Survival (95%CI)	European Monocentric Registry [11]	5-Year OS (%)	American SEER Registry [12]	5-Year OS (%)	Asian Monocentric Registry [13]	5-Year OS (%)
IA	1501	81.0	129.8 (129.8–133)			2170	77.5	618	94.8
IB	1095	68.5	112.8 (100.0-NA)	74	69.8	1065	63.3	313	89
IIA	1245	59.3	91.6 (79.1–103.1)			1241	50.8	345	84.6
IIB	1432	46.4	50.5 (46.6–58.2)	60	51.6	1404	35.3	455	76.1
IIIA	2310	30.5	25.0 (23.3–26.9)	30	25.9	2113	20.5	791	60.3
IIIB	1896	20.1	17.4 (16.4–18.7)	32	32.7	1466	13.5	920	40.9
IIIC	1067	8.3	11.8 (10.9–12.7)	28	9.8	735	5.3	825	27.5
IV	1449	5.6	8.9 (8.3–9.7)	17	4.5	0	NA	0	NA

Footnote: NCDB = National Cancer Database; SEER = Surveillance, Epidemiology and End Results; NA = not available; OS = Overall survival.

## Data Availability

Not applicable.
